# Transcriptome Analysis Reveals Sexual Disparities between Olfactory and Immune Gene Expression in the Olfactory Epithelium of *Megalobrama amblycephala*

**DOI:** 10.3390/ijms222313017

**Published:** 2021-12-01

**Authors:** Maolin Lv, Xiuli Chen, Xin Huang, Ning Liu, Weimin Wang, Han Liu

**Affiliations:** 1 Key Lab of Agricultural Animal Genetics, Breeding and Reproduction of Ministry of Education/Key Lab of Freshwater Animal Breeding, Ministry of Agriculture and Rural Affairs, College of Fisheries, Huazhong Agricultural University, Wuhan 430070, China; C764962149@163.com (M.L.); kuale303171@mail.hzau.edu.cn (X.H.); liuning1997@webmail.hzau.edu.cn (N.L.); wangwm@mail.hzau.edu.cn (W.W.); 2 Hubei Hongshan Laboratory, Engineering Research Center of Green Development for Conventional Aquatic Biological Industry in the Yangtze River Economic Belt, Ministry of Education, Wuhan 430070, China; 3 Guangxi Key Laboratory of Aquatic Genetic Breeding and Healthy Aquaculture, Guangxi Academy of Fishery Sciences, Nanning 530021, China; chenxiuli2001@163.com

**Keywords:** *Megalobrama amblycephala*, olfactory receptor, transcriptome analysis, immune system, fluorescence in situ hybridization

## Abstract

The olfactory organ is an important chemoreceptor in vertebrates. However, the sexual disparities in gene expression patterns in the olfactory organ in fish remain unstudied. Here, we conducted a transcriptome analysis of the olfactory epithelium (OE) of male and female blunt snout bream (Megalobrama amblycephala) to identify the differences. The histological analysis showed that there were 22 leaf-like olfactory lamellaes on one side of the OE of the adult blunt snout bream. The sensory area of OE is enriched with ciliated receptor cells and microvilli receptor cells. The transcriptome analysis showed that only 10 out of 336 olfactory receptor genes (224 ORs, 5 V1Rs, 55 V2Rs, and 52 TAARs) exhibited significant expression differences between males and females, and most of the differentially expressed genes were related to the immune system. We also validated these results using qPCR: 10 OR genes and 6 immunity-related genes significantly differed between males and females. The FISH analysis results indicated that the ORs were mainly expressed at the edge of the olfactory lamellae. Collectively, our study reveals that gender is not an important factor influencing the expression of olfactory receptors, but the expression of immune genes varies greatly between the genders in blunt snout bream.

## 1. Introduction

The olfactory organ is one of the most important sensory organs in vertebrates. Olfactory receptor genes are mainly expressed in the olfactory epithelium (OE) of the nasal cavity in fish. Olfactory receptors can distinguish a variety of odor molecules from the outside world, playing a crucial role in food selection, recognition of toxic substances, avoidance of natural enemies, and identification of individuals. At present, five receptor families have been identified in mammals, namely the main olfactory receptors (*ORs*) [1], vomeronasal type-1 receptors (*V1Rs*) [2], vomeronasal type-2 receptors (*V2Rs*) [3], trace amine-associated receptors (*TAARs*) [4], and formyl peptide receptors (*FPRs*) [5]. Each of these families contains a large number of genes. However, only four receptor families were identified in fish: *FPRs* have not been reported yet. The number of receptor genes in the four families in fish is far smaller than in mammals. For example, the number of functional *ORs* in African elephants is about 2000 [6], while that of *Pseudoliparis swirei* is only about 43 [7]. Since fish have no vomeronasal organ, *V1Rs* and *V2Rs* are called *ORAs* (olfactory receptors related to class A GPCRs) and *OlfCs* (olfactory receptors related to class C GPCRs), respectively. Among these four types of receptors, *ORs* mainly recognize water-soluble odor molecules and *TAARs* mainly recognize odorous molecules, while *V1Rs* and *V2Rs* recognize pheromones [8,9,10,11] and amino acid molecules [12,13,14].

The olfactory organ does not only recognize odor molecules: it is also one of the important immune organs in vertebrates and the main mucosal immune barrier against pathogens. Nasopharynx-associated lymphoid tissue was first described as the accumulation of pairs of lymphoids in the nasal cavity in rodents [15,16], and it was subsequently observed in mammals [17,18]. The olfactory organs of fish are constantly exposed to the presence of pathogens in the water. To protect the olfactory organs from pathogens in the water, the olfactory organs of teleost fish are rich in various white blood cells, scattered on the olfactory lamellae [19], which express innate and adaptive immunity-related molecules [20]. These white blood cells are adjacent to olfactory sensory neurons, supporting cells, and basal cells, forming a unique microenvironment and promoting neuro-immune communication.

Gender is a key factor affecting sense of smell in mammals. Early studies showed that female capabilities for the biological detection, identification, and discrimination of odors are better than those of the male [21]; subsequent electrophysiological experiments have also confirmed the advantages of females in terms of odor detection. In the behavioral response caused by olfactory signals, there are many obvious differences between adult male and female mice. For example, the male pheromone exocrine gland-secreting peptide 1 (ESP1) can regulate female reproductive behavior [22], while the specific signal protein (darcin) is inherent to female mice [23]. Sexual attraction and the fluctuation of sex hormones also affect the function of smell [24]. There are obvious differences in the expression of olfactory receptor genes between male and female rats, which may be the underlying reason for the differences in olfactory function between males and females. At present, the olfactory receptor genes in some fish species had been identified, such as *Megalobrama amblycephala*, *Danio rerio*, *Larimichthys crocea, Siniperca chuatsi*, etc. [25,26,27,28,29]. Previous studies about the impact of gender on smell mainly focused on the identification of sex pheromone. Sex differences in fish cause a different sensitivity to sex pheromone. Males are more specific in identifying sex pheromones, which means that these receptors must have a high degree of specificity [30]. However, the differences between male and female fish in gene expression patterns as well as the ability to detect food odor molecules remain unclear.

Blunt snout bream (*Megalobrama amblycephala*) is one of the most important farmed fish species in China. During the breeding process, we observed that females exhibit a faster growth rate than males. Olfaction is one of the main factors affecting feeding behavior, which may contribute to the growth differences between females and males. In the present study, we used the RNA-seq method to explore the differences in gene expression in OE of male and female blunt snout bream. We found that the OE of blunt snout bream expressed a large number of olfactory receptors, but the overall expression level did not show a huge difference between males and females. Interestingly, we observed that the OE simultaneously expressed a large number of immune genes and exhibited notable male vs. female differences in this aspect.

## 2. Results

### 2.1. The Structure of the Olfactory Epithelium

**Figure 1:**
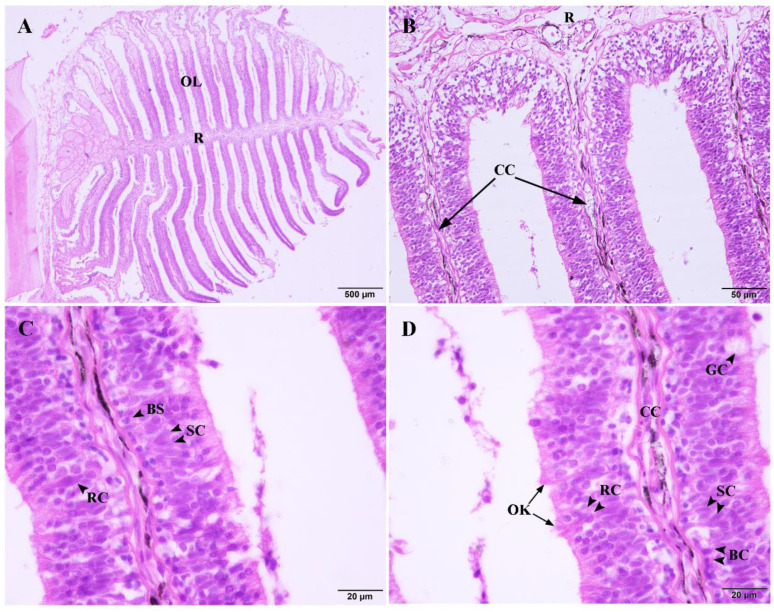
Semithin section of OE stained with H.E. (5 μm). (**A**) OE of blunt snout bream (40×). R, olfactory raphe; OL, olfactory lamellae. (**B**) Olfactory lamellae of blunt snout bream (400×). CC, central core (black arrow). (**C**,**D**) Olfactory lamellae of blunt snout bream (1000×). CC, central core; RC, olfactory receptor cell; GC, goblet cell; BC, basal cell; SC, support cell; OK, olfactory knob.

There were 22 leaf-like olfactory lamellaes (OL) radiating from the olfactory raphe (R) on one side of the olfactory tissue of the adult blunt snout bream (Figure 1A). The center of the olfactory lamellae was the central core (CC), which was composed of loose connective tissue and capillaries (Figure 1B). The OE is located on the surface of the olfactory lamellae and arranged on both sides of the CC. The cells of the OE are divided into three layers. Olfactory receptor cells (ORCs) are located in the outermost layer of the OE, and cilia or microvilli extend out of the OE. A large number of support cells (SCs) are tightly arranged and located in the middle layer of the OE. Basal cells (BCs) are located at the bottom of the OE and closed to the central core; Goblet cells (GCs) are inlaid and distributed throughout the OE (Figure 1C,D).

### 2.2. The SEM of the Olfactory Epithelium

**Figure 2:**
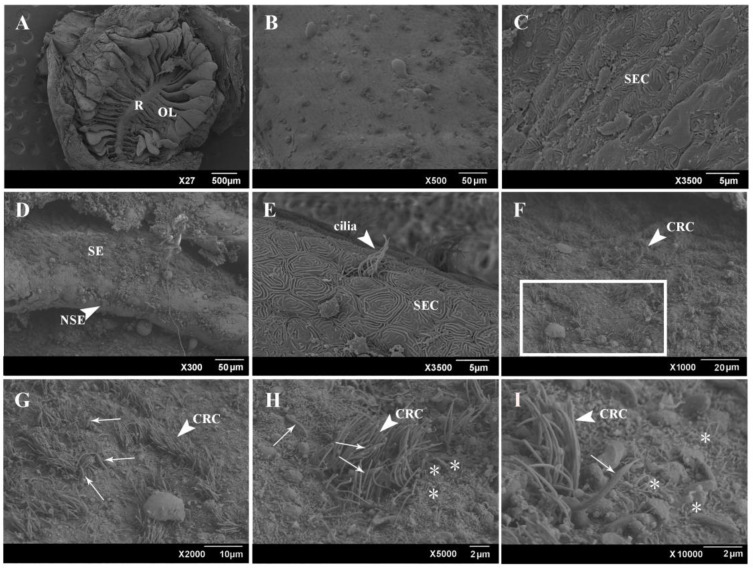
SEM of the general overview of OE. (**A**) General view showing OE that consisted of olfactory lamellae (OL) and olfactory raphe (R). (**B**) Higher magnifications of OE from A showing olfactory raphe. (**C**) Epithelium of olfactory raphe was consisted of stratified epithelial cells (SECs). (**D**) Sensory epithelium (SE) was distributed on the side of olfactory lamellae, and non-sensory epithelium (NSE, white arrow) was distributed on the margin of olfactory lamellae. (**E**) Epithelium of non-sensory epithelium (NSE) of olfactory lamellae consisted of stratified epithelial cells (SECs) and ciliated cells (white arrow). (**F**) Sensory epithelium (SE) of olfactory lamellae was covered with a large number of cilia. (**G**–**I**) Higher magnification of boxed area in F, showing ciliated receptor cells (short arrow), rod-shaped cilia (long arrow), and microvillous receptor cells (*).

The result of a scanning electron microscope (SEM) showed that the size and shape of the olfactory lamellae varied according to their position in the OE (Figure 2). The olfactory lamellae near the center had a larger volume. The median raphe was covered by stratified epithelial cells (SECs), with their apical surface provided by exhibited finger print-like microridges (Figure 2A–C). The sensory area is located on the lateral surface of the olfactory lamellae, whereas the non-sensory area was restricted to the margin. The cells in the non-sensory area mainly include lamellar epithelial cells and ciliated non-sensory cells (CNCs). The sensory area contained ciliated receptor cells (CRCs) and microvilli receptor cells (MRCs) that were distributed randomly. The ciliated olfactory receptor cells were dominant over the microvilli receptor cells (Figure 2D–F). The protruding parts of the dendrites of the microvilli receptor cells formed olfactory nodes, and dozens of microvilli of varying sizes grew at the olfactory nodes. In addition, rod-shaped cilia were mixed between ciliated cells and microvilli cells in the sensory area. The diameter of rod-shaped cilia was significantly larger than that of ordinary cilia, but the number was not large (Figure 2G–I).

### 2.3. Transcriptome Analysis

**Figure 3:**
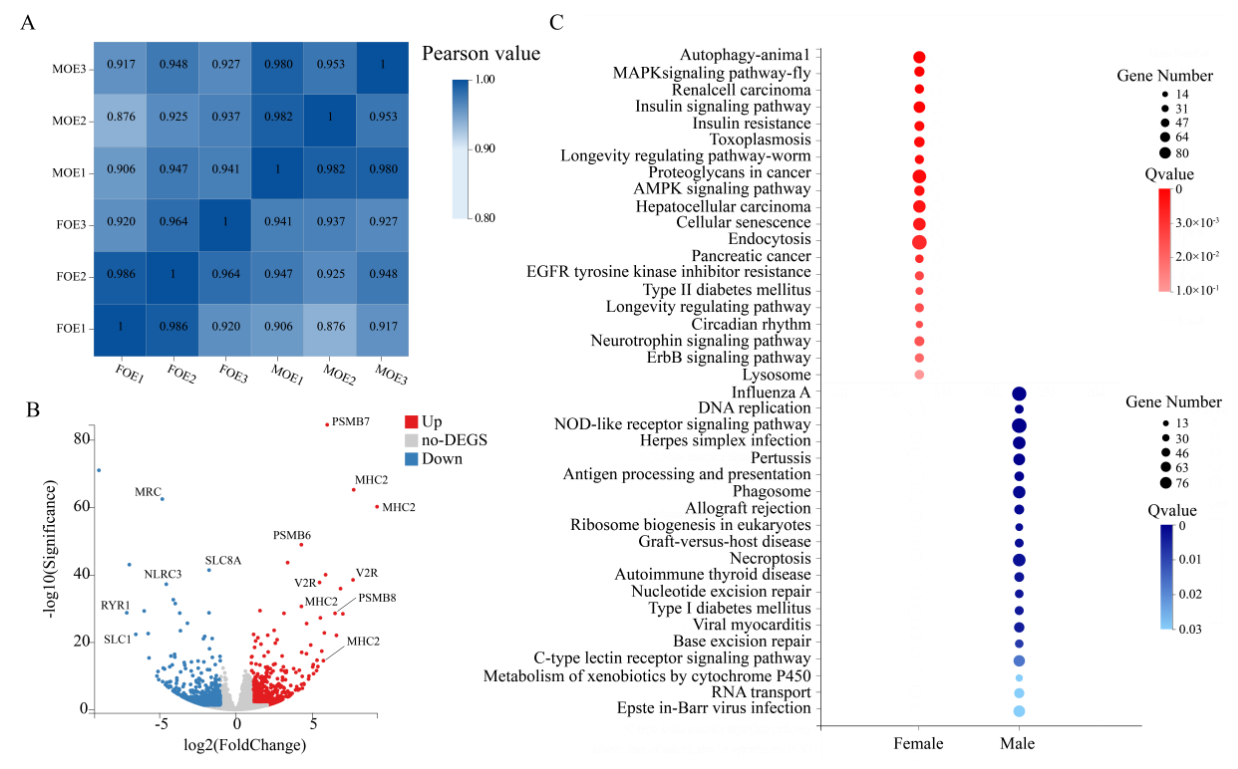
Sexual dimorphism in blunt snout bream olfactory system. (**A**) Heat map of cross-correlations of all samples using transcripts. (**B**) Log2-fold change between male and female samples is plotted against their −log10 FDR. (**C**) KEGG pathway enrichment of differentially expressed genes between OE of male and female blunt snout bream.

We evaluated the expression of genes among three biological replicate samples of both male and female. In general, the transcriptome profiles of the male OE and the female OE were highly correlated (Pearson values were ≥0.92) (Figure 3A, Supplementary Table S1). We deep-sequenced 40 Gb base pairs of RNA from six blunt snout bream OE samples (43.94 ± 0.56 s.d. million reads per sample) using RNA-seq. On average, 81.78% of clean reads mapped to the reference genome (*M. amblycephala*), 59.61% of which had unique mapping coordinates (Supplementary Table S2). We made a rough scale: FPKM < 1 corresponded to weak expression, FPKM > 10 to high expression, and FPKM values between 1 and 10 were classified as stable expression. In total, we annotated 30,271 protein-coding transcripts. Among these transcripts, 10,457 had a weak expression, 11,762 had a stable expression, and 8052 had a high expression.

### 2.4. Differentially Expressed Genes

To assess whether there are gender differences in genes expressed in the OE of blunt snout bream, we compared the gene expression between males and females. We found that the overall transcriptome profiles of the OE were highly similar between male and female blunt snout bream. We detected a total of 1717 transcripts showing higher expression levels in the male OE and 2137 transcripts showing higher expression levels in the female OE (Figure 3B, Supplementary Note S1, Supplementary Figure S1). Among these DEGs, 1162 genes were involved in immune and disease regulation. Genes from two families related to the immune system, the major histocompatibility complex (MHC) and the proteasome 20 s (PMSB), had the most significant differences in expression (Figure 3B).

To gain further insights into the biologic functions that differed between the male and female blunt snout bream OE, we performed a KEGG pathway enrichment analysis of the DEGs detected between them (Figure 3C, Supplementary Note S2). Compared with female fish, the up-regulated genes in male fish were mostly related to disease-related regulatory pathways, such as Proteoglycans in cancer (pathway ID: ko05205), Type II diabetes mellitus, Renal cell carcinoma, etc. The expression of genes involved in EGFR tyrosine kinase inhibitors (pathway ID: ko01521) and the ErbB signaling pathway (pathway ID: ko04012) was also up-regulated. Among the up-regulated genes in females, they were also mainly related to the immune system, such as the NOD-like receptors (NLRs, pathway ID: ko04621) and the C-type lectin receptors (CLRs, pathway ID: ko04625).

### 2.5. The Olfactory Receptor Repertoires

We identified the olfactory receptor genes in the transcriptome. Similar to zebrafish [31], the expression levels of olfactory receptor genes in the OE of blunt snout bream were relatively low. Only 10 of these genes were highly expressed. Among the 224 *OR*, 5 *V1R*, 55 *V2R*, and 52 *TAAR* genes, most of them had FPKM > 1, indicating that these olfactory receptor genes were mainly expressed and putatively functional in the OE tissue.

**Figure 4:**
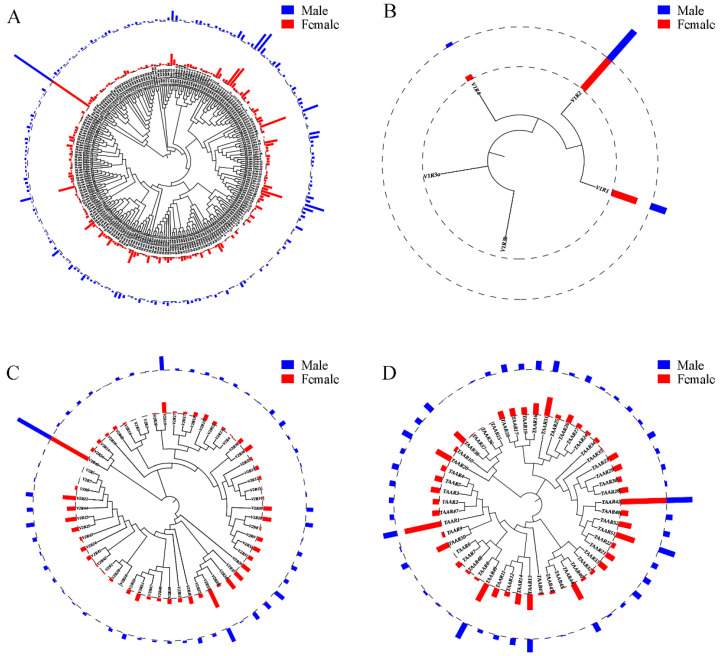
Expression pattern of chemosensory receptor genes in OE of blunt snout bream. Mean FPKM expression values across three samples between male (blue) and female (red) for *ORs* (**A**), *V1Rs* (**B**), *V2Rs* (**C**), and *TAARs* (**D**) genes. Phylogenetic tree was reconstructed using MEGA-X. Major clades had bootstrap values greater than 50% (1000 replicates).

To assess whether there are differences in the expression levels of olfactory receptor genes between the two sexes, we made several interesting observations. Firstly, the relative receptor abundance levels varied greatly among the different olfactory receptor genes (Figure 4). The top 48 genes accounted for half of the total expression, which is consistent with the results from humans [32] and mice [33]. Almost all of the olfactory receptor genes were not differentially expressed between the male and female blunt snout bream in the OE, except 5 *OR*, 4 *V2R*, and 1 *TAAR* genes. Interestingly, most of the olfactory receptor genes that exhibited sexual dimorphism had higher expression levels in females than in males.

### 2.6. Regulatory Network of Immune Genes

**Figure 5:**
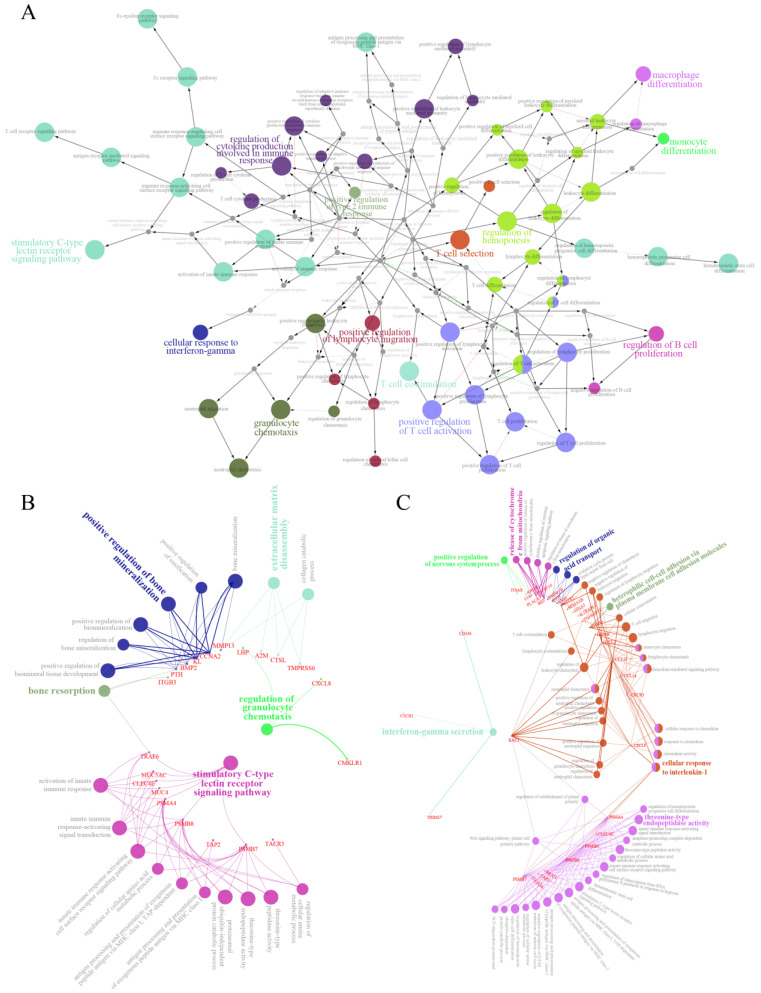
Gene co-expression network analysis. (**A**) Co-expression network of 3854 immune genes in transcriptome of blunt snout bream, where nodes indicate different pathways. (**B**) Co-expression network of top 100 immune genes expressed in females. (**C**) Co-expression network of top 100 immune genes expressed in males.

We detected 3854 genes classified as immune genes or related to the regulation of diseases, constructed a gene network map, and then used it to explore their role in the nasal mucosal immune system in blunt snout bream. The analysis revealed that signal pathways, such as T cell costimulation, B cell proliferation regulation, and C-type lectin receptor signaling pathway stimulation, dominate the nasal mucosal immune system of blunt snout bream (Figure 5A). Since the heat map indicated that immune genes exhibited the most prominent differences in expression levels (Supplementary Figure S2), we also performed a network analysis on the first 100 differentially expressed immune genes of male and female blunt snout bream (Figure 5B,C). All the key regulatory pathways showed obvious gender differences. In males, pathways such as regulation of granulocyte chemotaxis and stimulatory C-type lectin receptor signaling were dominant; whereas in females, interferon-gamma secretion and cellular response to interleukin-1 were dominant (Figure 5B,C).

### 2.7. Analysis of Differentially Expressed Transcription Factors

**Figure 6:**
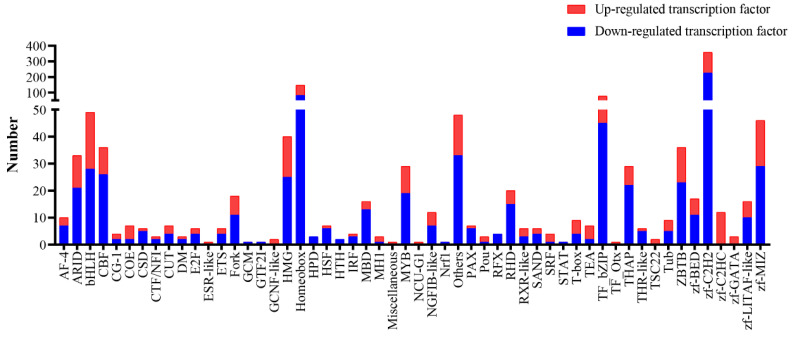
Differentially expressed transcription factor genes. Red boxes highlight the transcription factor families with large differences.

Based on the threshold of |log2FC| ≥ 0.25, differentially expressed non-redundant transcription factors were identified (Figure 6, Supplementary Note S3). Among them, 456 transcription factor genes were up-regulated, and 732 transcription factor genes were down-regulated in males. These differentially expressed genes were mainly the zinc finger C2H2 type (zf-C2H2), homeobox, basic region-leucine zipper (bZIP), and other transcription factor families.

### 2.8. Quantitative Real-Time PCR (qPCR)

**Figure 7:**
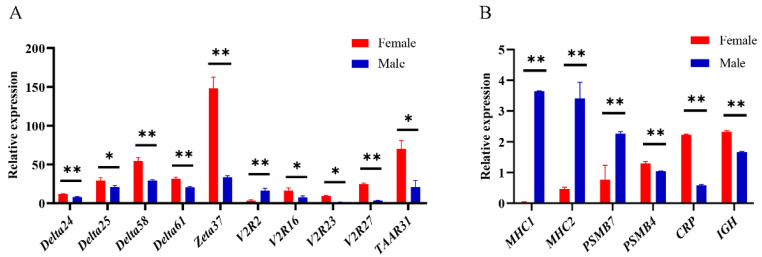
Expression analysis of differentially expressed genes. (**A**) Quantitative RT-PCR analysis of differentially expressed olfactory receptor genes. (**B**) Quantitative RT-PCR analysis of differentially expressed immune genes. Statistically significant differences from the males and females are marked as * *P* < 0.05 and ** *P* < 0.01.

In order to verify the expression results from RNA-seq, we further employed qPCR to measure relative mRNA levels for 16 candidate genes. The efficiency of primers was 91–110% (Supplementary Figures S3 and S4). Our results demonstrated that the expression patterns for these genes were highly consistent between RNA-seq and qPCR (Figure 7, Supplementary Note S4), which confirms the reliability of our RNA-seq results. The 10 olfactory receptor genes, except for *V2R2*, showed higher expression in females than in males.

### 2.9. Fluorescence In Situ Hybridization

**Figure 8:**
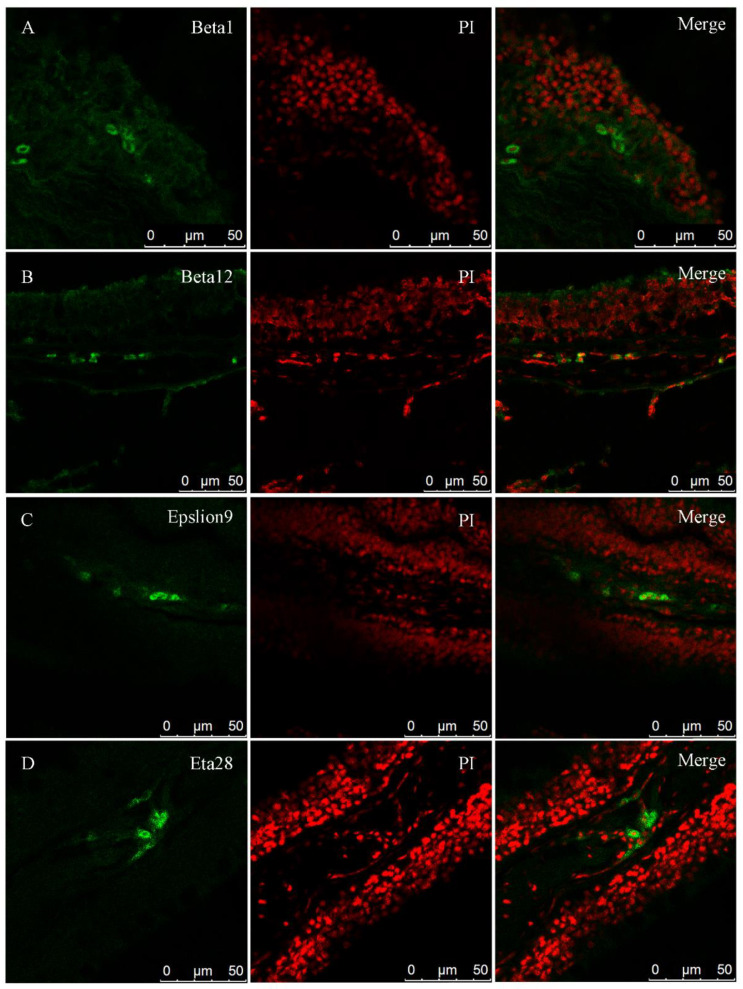
Fluorescence in situ hybridization of four genes (*beta1*, *beta12*, *epsilon9*, and *eta28*). Green color stained cells are those that express the relevant genes (labeled with Anti-DIG-POD, Fab fragments), whereas nuclei are stained red with propidium iodide.

Each sensory neuron (ORN) expresses only one kind of olfactory receptor in many different vertebrates [34,35], so we hypothesized that the same might be true in blunt snout bream. Due to a large number of olfactory receptor genes and the high degree of similarity between them, we selected four genes among the *ORs* (*beta1*, *beta12*, *epsilon9,* and *eta28*) as representatives for the fluorescence in situ hybridization analysis (Figure 8). As we expected, the cells expressing these four genes were mainly distributed near the surface of the olfactory layer, but it is impossible to determine whether they were expressed in ciliated receptor cells or microvilli receptor cells.

## 3. Discussion

Fish evolved highly developed chemoreceptors to adapt to the complex environment in the water. The olfactory organ, as a chemoreceptor that mediates foraging, information exchange, and reproductive behavior between individuals, has special significance for fish. A previous study in mammals indicated that gender is one of the factors that affect olfactory sensitivity [21,36]. However, the transcriptome of zebrafish did not show differences between the two sexes [31]. In this study, we observed the structure of the olfactory epithelium tissue and used the RNA-seq method to explore the differences in gene expression in the OE of male and female blunt snout bream. We found that the OE of blunt snout bream expressed a large number of olfactory receptors, but the overall expression levels did not show a huge difference between males and females. However, a large number of immune genes exhibited notable male vs. female differences.

The olfactory lamellae of blunt snout bream were arranged symmetrically on both sides of the long olfactory raphe, laterally or obliquely. They belonged to the G type, which indicated that the olfactory system of blunt snout bream was relatively highly developed [37]. The number of olfactory lamellae in adult blunt snout bream was 22 to 24 on one side. A previous study indicated that more olfactory lamellae could provide a large surface area to receive the stimulation of external odor molecules, pheromones, and other signals [38]. The sensory epithelium and non-sensory epithelium of blunt snout bream were randomly distributed on the surface of a single lamella, which exhibited an intermittent distribution [39]. This suggested that blunt snout bream might have a strong sense of smell and mainly rely on that sense of smell for feeding and other activities.

Many reports on *Homo sapiens* show that females perform comparatively better in odor recognition tests [21,39]. That is, females have a higher threshold of sensitivity to various compounds. Doty and Cameron proposed that the advantage of female olfactory perception was related to the complex interaction between neuroendocrine factors and the olfactory system [24]. However, changes in hormone levels, such as estrogen, also have an impact on olfaction. A previous study has demonstrated that the fluctuation of sex hormones affects the threshold-level of olfactory sensitivity [36]. Some functional studies have identified several olfactory receptor ligands in fish. For example, in zebrafish, *or114-1* and *or114-2* are receptors for the sex pheromone prostaglandin F2α, which regulates the courtship behavior of male zebrafish [40]. In our study, we found no obvious gender differences in the expression of olfactory receptors in blunt snout bream. Only 10 out of 336 olfactory receptor genes (5 *ORs*, 4 *V2Rs*, and 1 *TAAR*) were expressed differentially between the two sexes, and most of them exhibited relatively small differences. Therefore, it is unlikely that the different behaviors of males and females in response to some chemical pheromones can be solely accounted for by transcriptional differences in blunt snout bream. *V1Rs* are thought to be related to the recognition of pheromones and may be involved in reproductive behavior [11]. We detected that the expression levels among five *V1R* genes were different. However, there was no difference between male and female, which was consistent with the results in rabbits [41].

Our fluorescence in situ hybridization results for four *ORs* showed that each gene was only expressed in a few cells, which may be related to the expression of only one receptor gene. Once OSNs select a certain olfactory receptor gene, it will be stably expressed throughout the life cycle of the cell, and all other olfactory receptor genes in the genome will be silenced [42,43]. How these cells select the expressed genes is still unclear. It might be related to the control of certain cis-acting regulatory elements [44]. The expression of *ORs* mainly occurs on the olfactory lamellae, close to the surface. This is exactly where the cilia receptor cells are located. Perhaps, as in other fishes, the *ORs* of blunt snout bream are mainly expressed in cilia receptor cells, but this hypothesis needs to be verified through further experiments.

The OE is one of the immune organs in fish, which is confirmed by the detection of a large number of immune genes in the olfactory epithelial cells of blunt snout bream. Although the effects of sex on the immune system have been reported in humans [45,46,47], birds [48,49], fruit flies [50], and other species [51], there are few reports on fish. We found that the nasal mucosal immune system of blunt snout bream showed obvious gender differences in the expression of genes such as MHC, IL, and others. A previous study has demonstrated that there are some differences in the nature and intensity of the immune response between males and females, and there are also gender differences in the prevalence of malignant tumors, autoimmune diseases, and infectious diseases [52]. Although females usually have a stronger immune response to certain malignant tumors, they are also more likely to suffer from inflammation [21]. This is consistent with our detection of higher expression of genes related to the infectious A signaling pathway and NOD-like receptors signaling pathway. Studies have shown that males are at greater risk of cancer, and we also found corresponding indications when we analyzed differential gene expression between female and male fish. Males exhibited up-regulation of some genes associated with cancers [53,54], such as kidney cancer and pancreatic cancer, while females did not. There is ample evidence that in invertebrates, humoral immunity and cell-mediated immune response are more effective in females than in males [55]. For example, under antigen stimulation, the circulating levels of immunoglobulin for females are far greater than those of males [56], making the female immune response stronger and longer lasting [57]. In mammals, female-derived antigen-presenting cells (APC) present peptides more efficiently than male-derived antigen-presenting cells (APC) [58]. Similarly, genes related to antigen presentation and processing in female bream also showed an up-regulation trend. In insects, lizards [51], birds [48], and mammals [59], the innate and adaptive immune responses of males are generally lower than in females. Our network analysis of differentially expressed genes also produced a congruent result. That is to say, in females, the up-regulated genes are mainly related to the innate immune response, the antibacterial humoral response, and the humoral immune response, which is not the case in males.

## 4. Materials and Methods

### 4.1. Sample Collection

**Figure 9:**
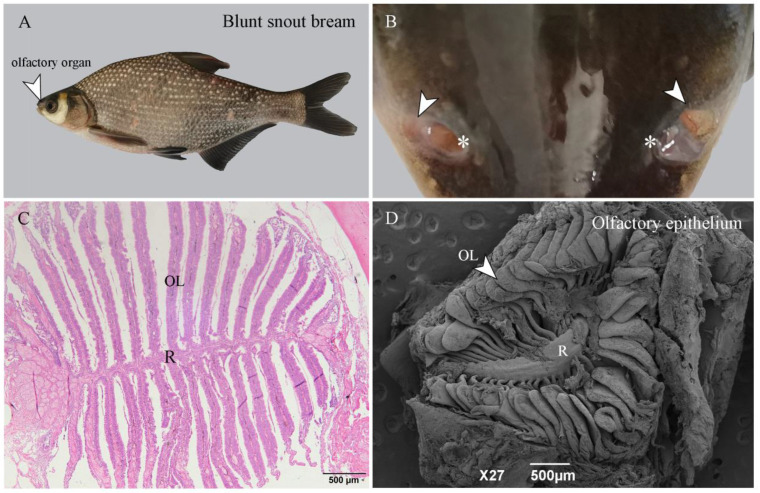
OE of blunt snout bream. (**A**) Blunt snout bream specimen. (**B**) Location of OE (arrows) and anterior nostrils (*) on blunt snout bream. (**C**) H.E. staining of OE of blunt snout bream. R: olfactory raphe; OL: olfactory lamellaes. (**D**) Scanning electron microscopy of OE of blunt snout bream.

Fishes used in this study were obtained from the Nanhu breeding base of the Fisheries College of Huazhong Agricultural University, China. All experiments were performed following the guidelines approved by the Animal Use and Care Committee of Huazhong Agricultural University, China (HZAUFI-2018-014). After the fishes were anesthetized, scissors were used to cut the flaps between the front and back nostrils to expose the OE. Then, we used a scalpel to cut out the OE. The olfactory epithelium from six sexually mature (2-year-old) blunt snout breams was dissected (Figure 9) and stored in 4% paraformaldehyde and 2.5% glutaraldehyde (three samples each) for hematoxylin-eosin staining (H.E. staining) and scanning electron microscopic (SEM) observation, respectively. In addition, three independent biological replicates of male and female olfactory epithelium samples were prepared and immediately frozen in liquid nitrogen and then long-term stored at −80 °C before RNA extraction.

### 4.2. RNA Extraction and BGISEQ Library Construction

The total RNA was extracted from the olfactory epithelium of each sample (~100 mg) of blunt snout bream using a RNAiso Plus Kit (Takara Bio, Beijing, China, code No. 9109) in accordance with the instructions of the manufacturer. The overall quality of RNA was assessed using Agarose Gel Electrophoresis. Subsequently, total RNA was also qualified and quantified using the 2100 Bioanalyzer system (Agilent Technology, CA, USA). All samples that met the quality requirements were used as the RNA-Seq library preparation.

Oligo(dT)-attached magnetic beads were used to purify the mRNA. Purified mRNA was fragmented into small pieces with fragmenting buffer before reverse transcription was conducted by using random N6 primers, which then went through some steps (Supplementary Figure S5). After that, the raw reads were obtained from BGISEQ-500.

### 4.3. Analysis of RNA-Seq Data

The raw sequencing data containing low-quality reads, linker contamination, and high N content of unknown bases were removed before data analysis to ensure reliability. After obtaining the clean reads, we used HISAT to align the clean reads to the reference genome [60]. Bowtie2 was used to align clean reads to the reference genome (PRJNA343584) [26], and RSEM was used to calculate the gene expression level of each sample [61,62]. Based on the principle of negative binomial distribution differential expression genes (DEGs), analysis was applied to identify differentially regulated genes according to the method described in Love et al. [63]. We use difference multiples of twice or more and a corrected *P* value of less than 0.05 to screen for differential genes. Detailed information about transcription factors was obtained by comparing with AnimalTFDB 2.0 [64]. The R packages “pheatmap” and “EnhancedVolcano” were used to plot heatmaps and volcano plots, respectively. Gene Ontology (GO) and Kyoto Encyclopedia of Genes and Genomes (KEGG) pathway enrichment analyses were performed using the DAVID Bioinformatics Resources 6.8 [65,66].

### 4.4. Data Mining for the Olfactory Receptor Genes

We extracted all sequences of annotated and automatically predicted paralogs of the *OR*, *V1R*, *V2R*, and *TAAR* genes from the blunt snout bream genome. We only considered a gene as a putative chemosensory receptor gene for a given family after confirming the evolutionary relationships of candidates within each chemosensory receptor family clade via a phylogenetic analysis.

### 4.5. Gene Network Analyses

The ClueGO module of the Cytoscape software [67] was used to construct a network interaction map of immune genes to find the key regulatory pathways of the immune system in the OE tissue of blunt snout bream. We also constructed a network interaction map of the immune genes that differentially expressed in males and females, to explore the effect of the impact of the sex of blunt snout bream on the differential expression of immune system genes.

### 4.6. Quantitative Real-Time PCR (qPCR)

In order to confirm the differentially expressed genes detected by RNA-seq, we further applied quantitative real-time PCR (qPCR) to a subset of genes significantly differentially expressed between the olfactory epitheliums of male and female blunt snout bream. The NCBI primer tool (https://www.ncbi.nlm.nih.gov/tools/primer-blast/ (accessed on 20 September 2020)) was used to design primers for these genes (Supplementary Tables S3 and S4). We used the PrimeScript RT Reagent Kit with gDNA Eraser (TaKaRa, code No. RR047A) to synthesize the cDNA. qRT-PCR experiments were performed using TB Green^®^ Premix Ex Taq™ II (Tli RNaseH Plus) (TaKaRa, code No. RR820A) by the QuantStudio^TM 6^ Flex qRT-PCR system (ABI, Foster City, CA, USA). Thermal cycling conditions were for 5 min at 95 °C, then 40 cycles at 95 °C for 20 s, and at 60 °C for 25 s. Using the *β-actin* (Supplementary Tables S1 and S2) of blunt snout bream as a reference gene, we used the comparative 2^-^^△△C^^t^ method [68] to determine the relative gene expression between the OE of male and female blunt snout bream. IBM SPSS Statistics 22.0 software was used to perform a Student’s t-test to compare the differences between the two groups [69]. *P* < 0.05 (significant) and *P* < 0.01 (highly significant) were considered to indicate statistical significance. The fold change values were the average of three biological replicates in each group.

### 4.7. Fluorescence In Situ Hybridization

The OE of blunt snout bream was fixed with 4% paraformaldehyde for 24 h, dehydrated by a series of gradient ethanol baths, embedded in paraffin, and then sectioned (5 µm). Fragments (length was between 150 and 250 bp) of four genes (*beta1*, *beta12*, *epsilon9,* and *eta28*) were cloned using the primers shown in Supplementary Table S5. The PCR products were purified, and then the in situ hybridization probe was synthesized in accordance with the instructions of the manufacturer, Sigma-Aldrich, using the DIG RNA Labeling Kit (SP6/T7) (Roche Diagnostics GmbH, Mannheim, Germany, SKU: 11175025910). The steps of fluorescence in situ hybridization were performed according to the method described in Alamri et al. [70]. Anti-DIG-POD, Fab fragments (Roche Diagnostics GmbH, SKU: 11207733910) from Sigma-Aldrich were used as antibodies.

## 5. Conclusions

In this study, we studied the OE of blunt snout bream by using histology and SEM. Furthermore, RNA-Seq-based transcriptomic analysis was used to investigate the differences in gene expression in the OE between male and female blunt snout bream. We found that most of the olfactory receptor genes are expressed in a similar pattern in the OE of blunt snout bream, and a few genes showed sex-specific differences. However, in the nasal mucosal immune system, gene expression showed obvious differences between males and females, indicating that gender was an important factor affecting immune gene expression. This study provides a good reference for targeted research on the female and male immune systems in blunt snout bream.

## Data Availability

Datasets supporting the results of this article were deposited in the NCBI Sequence Read Archive (SRA) database and the accession number is PRJ NA 781150.

## Supplementary Materials

The following are available online at https://www.mdpi.com/article/10.3390/ijms222313017/s1.
