# Supplementary material for: Transcriptome Analysis Reveals Sexual Disparities between Olfactory and Immune Gene Expression in the Olfactory Epithelium of Megalobrama amblycephala
Source: Int J Mol Sci. 2021 Dec 1;22(23):13017. doi: 10.3390/ijms222313017 (PMC8658043; doi:10.3390/ijms222313017)
Supplement: Supplementary file 1 [file ijms-22-13017-s001.zip › ijms-1448737-supplementary.pdf]

Supplementary Table S1. Correlations of all samples

Samples	FOE1	FOE2	FOE3	MOE1	MOE2	MOE3
FOE1	1	0.985947	0.920276	0.905899	0.876285	0.917336
FOE2	0.985947	1	0.963644	0.946915	0.924795	0.948175
FOE3	0.920276	0.963644	1	0.940717	0.937488	0.926511
MOE1	0.905899	0.946915	0.940717	1	0.981587	0.980111
MOE2	0.876285	0.924795	0.937488	0.981587	1	0.95272
MOE3	0.917336	0.948175	0.926511	0.980111	0.95272	1

Supplementary Table S2. Summary of RNA-seq data

Sample	Total clean reads (Mb)	Total clean bases (Gb)	Clean reads Q20 (%)	Total mapping genome ratio (%)	Uniquely mapping genome ratio (%)
FOE1	43.98	6.60	96.86	82.06	58.40
FOE2	43.45	6.52	96.87	81.55	58.29
FOE3	43.10	6.47	96.92	81.65	58.53
MOE1	44.49	6.67	97.26	82.11	61.37
MOE2	44.26	6.64	97.11	81.28	60.04
MOE3	44.38	6.66	97.12	82.01	61.02

Supplementary Table S3. The qRT-PCR primers for differentially expressed olfactory receptor genes in *M. amblycephala*

Gene name	Sequence (5'—3')	Product size (bp)
OR-Zeta37-F	ACGCACCAGTGGAATTTTGC	172
OR-Zeta37-R	TGGCCACTAACATGAGGTGT	
OR-Delta24-F	TTTGCCACGCTTATTGCAGG	179
OR-Delta24-R	TTGCCGTGTATCGCAATGGA	
OR-Delta25-F	CTCACTGGGGTGGGTGTTAC	198
OR-Delta25-R	CCCCCGAACTTCCTAGACTC	
OR-Delta58-F	ACTGAAGACCTGCACACCAC	191
OR-Delta58-R	TCAGAGCCTGAACACTCAGC	
OR-Delta61-F	CCCTCTGAGCGCTACATCAC	186
OR-Delta61-R	GGCTCCTGCCGACAGTTTTA	
V2R2-F	CACGTTTTGCCCAGGCTTAC	194
V2R2-R	ATACGGGGTTGTGTGTGACC	
V2R16-F	CGCCAACCAAAAGGATTGGG	172
V2R16-R	GTAGGCGGTGACAAGGTTCA	
V2R23-F	ACAGCACTGGTGCCACAATA	126
V2R23-R	GCACAAATCGTTGGTCAGGG	
V2R27-F	GAGAGGGTTCTGCTCACGG	146
V2R27-R	ATGTGGGCGAGCTCGATAAT	
TAAR31-F	GACTGAGAGGCAGCATTGGT	196
TAAR31-R	GGAGTCCAGCTGTTTCTGTGA	
β-actin-F	ACCCACACCGTGCCCATCTA	152
β-actin-R	CGGACAATTTCTCTTTCGGCTG	

Supplementary Table S4. The qRT-PCR primers for differentially expressed immunity-related genes in *M. amblycephala*

Gene name	Sequence (5'—3')	Product size (bp)
MHC2-F	GTATCGCCCAAATGATGACGG	170
MHC2-R	AGAACACAGCTGGACCAACA	
PSMB7-F	AGGCACTGGTAAGTGATGCC	124
PSMB7-R	GGGGACTCTCTGTGAGGTCT	
PSMA4-F	GAAGTTCCAGGTGCCCGTTA	195
PSMA4-R	AGTTTCCATGGCAGAGTCCG	
CRP-F	TACTCTCTGCATGCGTGTCG	148
CRP-R	AAATGCTCCATCGCCACTGA	
IGH-F	CAGGCACCTGGAAAAGGACT	156
IGH-R	CACCGCAGTGTCTTCAGTCT	
MHC1-F	ACCAAACACAAATGGGAGGC	180
MHC1-R	AGCATGACACGTCACTGGAG	
β-actin-F	ACCCACACCGTGCCCATCTA	152
β-actin-R	CGGACAATTTCTCTTTCGGCTG	

Supplementary Table S5. The primers used for fluorescence *in situ* hybridization probe synthesis

Gene name	Sequence (5'—3')	Product size (bp)
Beta1-F	ACCATAGTCTCTCTGGCTGGA	156
Beta1-R	TAATACGACTCACTATAGGGGGCCTTTATTTGGGCCTTACC	
Beta12-F	TGGCCTGATCGCAACTTTGG	248
Beta12-R	TAATACGACTCACTATAGGGCCCAAGCAAGATGGTGGACT	
epsilon9-F	GGGCATACCCTCTCCCTTAC	221
epsilon9-R	TAATACGACTCACTATAGGGCAGTCTTCCTTGAGCGCTTG	
eta28-F	CTTCAGGGACACACGCTACA	212
eta28-R	TAATACGACTCACTATAGGGATATCGCCACATAGCGCTCC	

Supplementary Figure S1

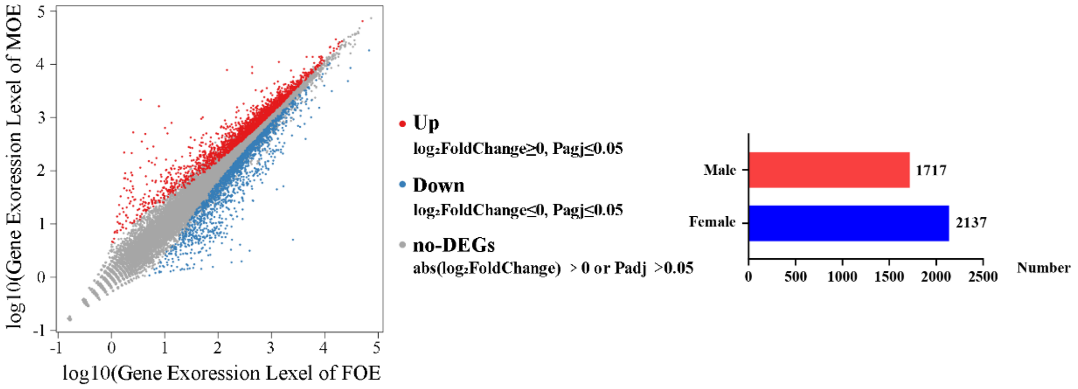

Figure S1. The number of DEGs. Red indicates genes that are up-regulated in males; Blue indicates genes that are up-regulated in females.

Supplementary Figure S2

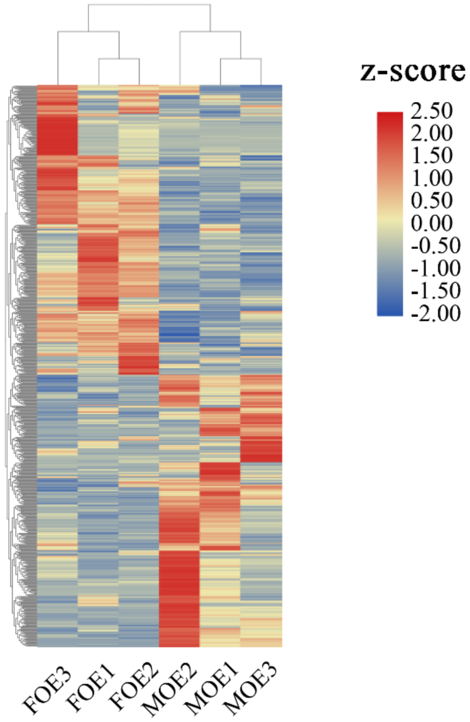

Figure S2. Heatmap of immunity-related genes. Red indicates genes up-regulated in males; Blue indicates genes up-regulated in females.

Supplementary Figure S3

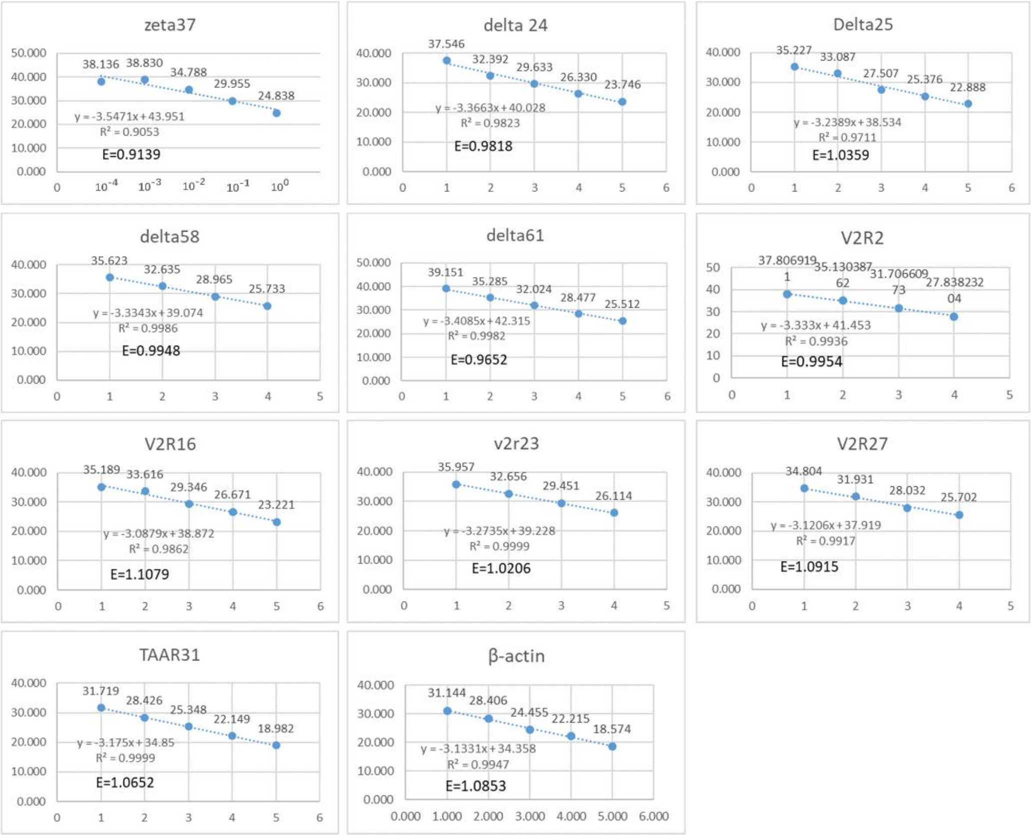

Figure S3. The efficiency of the olfactory receptor genes primers. Standard curves made by serial dilution of cDNA of OE. R^2 represents the correlation coefficient, and "E" represents the efficiency.

Supplementary Figure S4

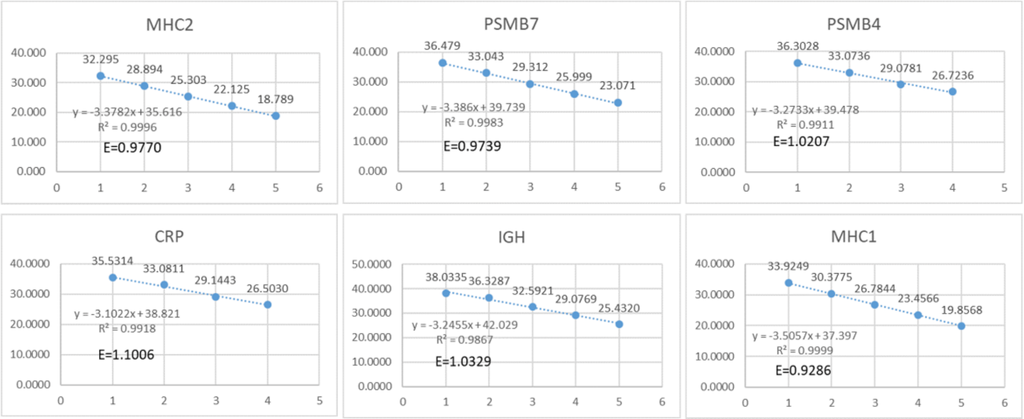

Figure S4. The efficiency of primers for immune genes. Standard curves made by serial dilution of cDNA of OE. R2 represents the correlation coefficient, and "E" represents the efficiency.

Supplementary Figure S5

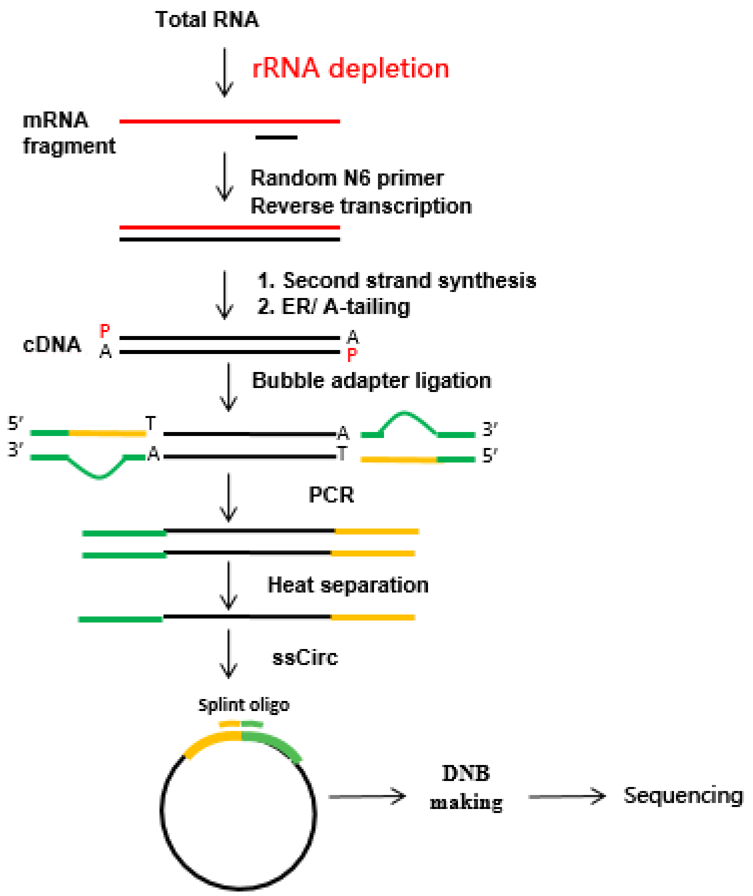

Figure S5. Flow chart of mRNA library construction.
